# A prospective study of breast anthropomorphic measurements, volume and ptosis in 605 Asian patients with breast cancer or benign breast disease

**DOI:** 10.1371/journal.pone.0172122

**Published:** 2017-02-13

**Authors:** Nai-si Huang, Chen-lian Quan, Miao Mo, Jia-jian Chen, Ben-long Yang, Xiao-yan Huang, Jiong Wu

**Affiliations:** 1 Department of Breast Surgery, Fudan University Shanghai Cancer Center, Shanghai, China; 2 Department of Oncology, Fudan University, Shanghai Medical College, Fudan University, Shanghai, China; 3 Department of Clinical Statistics, Fudan University Shanghai Cancer Center, Shanghai, China; 4 Collaborative Innovation Center of Cancer Medicine, Shanghai, China; di Pompeo d'Illasi, Universita degli Studi di Roma La Sapienza Facolta di Medicina e Psicologia, ITALY

## Abstract

**Objectives:**

The current study aims to summarize breast anthropomorphic measurement features in Chinese patients with breast diseases and to investigate their potential correlations with demographic factors.

**Materials and methods:**

Fifteen breast anthropomorphic parameters of 605 Chinese female patients were collected prospectively. Breast ptosis status was scaled by two methods and breast volume was calculated according to a modified formula of BREAST-V.

**Results:**

Among 1210 breasts, the average breast volume was 340.0±109.1 ml (91.8–919.2 ml). The distance from the nipple to the inframammary fold was 7.5±1.6 cm in the standing position. The width of the breast base was 14.3±1.4 cm (8.5–23.5 cm). The incidence of breast ptosis was 22.8% (274/1204), of which 37 (23.5%) and 79 (31.7%) women had severe ptosis assessed by different criteria. Increased height (OR[odds ratio] = 1.500, *P*<0.001), post-menopausal status (OR = 1.463, *P* = 0.02), increased BMI, breastfeeding for 7–12 months (OR = 1.882, *P* = 0.008) and more than one year (OR = 2.367, *P* = 0.001) were risk factors for an increased breast volume. Post-menopausal status (OR = 2.390, *P*<0.001 and OR = 2.621, *P*<0.001 for different scales), BMI≥24.7 kg/m2 (OR = 3.149, *P*<0.001 and OR = 2.495, *P* = 0.002), breastfeeding for 7–12 months (OR = 4.136, *P* = 0.004 and OR = 4.010, *P* = 0.002), and breastfeeding for more than one year (OR = 6.934, *P*<0.001 and OR = 6.707, *P*<0.001) were independent risk factors for breast ptosis.

**Conclusions:**

The current study provides anthropomorphic measurements data of Chinese women with breast diseases, which are useful for cosmetic and reconstructive breast surgery decisions. Post-menopausal status, increased BMI, and breastfeeding for more than six months were independent risk factors for both increased breast volume and breast ptosis.

## Introduction

Preoperative assessment of breast features is indispensable for both cosmetic and reconstructive breast surgical procedures[1]. The predicted resection or implanting volume required for breast reduction or augmentation can provide surgeons with practical and reproducible information during preoperative planning and in their cosmetic practice[2]. Regarding reconstructive surgery, which is increasingly desired by patients with breast cancer after mastectomy, an accurate determination of breast volume is a necessity to achieve bilateral symmetry[3]. Objective breast measurement parameters can also aid in the location and design of the nipple-areolar reconstruction. In some cases, additional plastic surgery on the contralateral side can help to achieve an aesthetically balanced profile with the guidance of breast measurement data[4].

Requests for reliable breast features date back to the 1970s. Since then, numerous methods of evaluation have been developed. However, among the available studies, reports derived from Anglo-American and European populations have been far more abundant than those for Asian populations[5]. Although reports of Asian breast features are available, most of them were conducted in young women or patients undergoing plastic surgery and, therefore, are not applicable to patients treated with mastectomy[6]. Because mastectomy remains the primary strategy for the treatment of early-stage breast cancer in China[7], post-mastectomy reconstruction is of great importance in the Chinese population. The lack of correlative studies has resulted in an urgent need for databases with information that is pertinent to Asian breast cancer patients.

The current study prospectively admitted patients with malignant and benign breast diseases for surgery in a single center. Breast feature information was collected using reliable and reproducible anthropomorphic measurements, and the breast volume was calculated according to a modified formula of BREAST-V (a latest validated formula)[2]. The purpose of the current study was to summarize breast measurements, volume and ptosis in Asian patients with breast diseases and to investigate potential correlations between demographic factors and breast features.

## Materials and methods

### Patients

A total of 605 consecutive patients who were admitted for breast surgery from July 2014 to October 2014 in the Department of Breast Surgery at Fudan University Shanghai Cancer Center, were prospectively admitted in this study. Patients who had undergone prior major breast surgery (mastectomy, breast-conserving surgery, breast reconstruction, breast reduction, and breast augmentation) were excluded. However, patients who received minor breast surgery (open biopsy, mammotone biopsy and core needle biopsy) without changes of breast appearances were included. Written informed consent was obtained from each patient, and the protocol was approved by the Ethics Committee of Fudan University Shanghai Cancer Center.

### Breast measurements

Seven nurses were trained to obtain breast measurements. For four months, 1210 breasts of 605 Chinese female patients were measured. Fifteen anthropomorphic parameters were collected for each breast: chest circumference under the axilla (CCA), chest circumference across the nipple (CCN), sternal notch-nipple distance (SN-N), clavicle-nipple distance (C-N), nipple-inframammary fold distance (N-IMF), stretched nipple-inframammary fold distance (SN-IMF), breast base width (BBW), nipple-sternal distance (N-S), breast-breast distance (B-B), breast projection (BP), areolar diameter on the horizontal level (ADH), areolar diameter on the vertical level (ADV), nipple projection (NP), nipple diameter (ND), and nipple-inframammary fold distance on the lateral view (LN-IMF).

Breast ptosis status was scored based on the criteria reported by Regnault P (RP scale)[8] as follows: Grade I, mild ptosis—the nipple descends to the level of the inframammary fold; Grade II, moderate ptosis—the nipple falls below the inframammary fold but remains above the lowest contour of the breast; Grade III: severe ptosis—the nipple is below the inframammary fold and reaches the lowest contour of the breast. We also used LaTrenta and Hoffman’s scale (LH scale)[9] to score breast ptosis according to the LN-IMF distance.

BREAST-V is a validated breast volume formula reported by Longo et al.[2]. We modified BREAST-V because one of the variable FF_p_, could not be precisely measured in our study population since the proportion of Chinese women with breast ptosis is far less than that in American or European countries. In that case, we used BP to replace FF_p_ in the BREAST-V formula to estimate breast volume in our cohort as follows: breast volume = −231.66 + 0.5747 × (SN-N)^2^ + 18.5478 × (BP) + 14.5087 × (N-IMF).

### Statistical analysis

The mean, standard deviation (SD) and range were reported for each measured data point. Univariate and multivariate ordinal logistic regression analysis were conducted to explore the correlation between demographic factors and breast volume and ptosis. The mean breast volume was calculated on a per patient basis. Breast volume was categorized by quartile, whereas breast ptosis was categorized by the RP and LH scales. Two-tailed *P* values were adopted, and *P*<0.05 was considered statistically significant. All variables with *P* values <0.1 in univariate analysis were included into multivariate analysis. Statistical analyses were performed using SAS version 9.2.

## Results

### Baseline characteristics of the patients

Among 605 female patients, 458 (75.7%) had malignant breast tumors, five had borderline tumors and 142 had benign breast disease on final pathology. The average age was 48.8±11.7 years, and the average BMI was 22.9± 3.0 kg/m^2^. More than half of the patients (57.2%) were pre-menopausal, and only 5.5% had no deliveries, whereas 29.1% patients had a history of two or more deliveries. The majority (70.1%) of the patients had a history of breastfeeding for more than 6 months (Table 1).

**Table 1:** Baseline characteristics of all patients.

Parameters	N	%
Age (mean±SD)	48.8±11.7	
Height (cm, mean±SD)	159.6±5.0	
BMI (mean±SD)	22.9±3.0	
Menopausal status		
Pre-menopausal	346	57.2
Post-menopausal	259	42.8
Literacy		
Primary school and below	114	18.8
Middle school	273	45.1
College and above	148	24.5
Not available	70	11.6
Number of deliveries		
None	33	5.5
One birth	396	65.5
Two or more births	176	29.1
Breastfeeding (months)		
None	85	14.0
1–6 months	96	15.9
7–12 months	244	40.3
≥13 months	180	29.8
Pathology		
Malignant	458	75.7
Borderline	5	0.8
Benign	142	23.4

SD, standard deviation; BMI, body mass index.

For patients with malignant disease, 41 were *in situ* carcinoma, among which 37 were ductal carcinoma *in situ* (DCIS), two were lobular carcinoma *in situ* (LCIS), and two were Paget’s disease. Of 417 cases of invasive disease, the majority (367/417, 88.0%) were invasive ductal carcinoma (IDC), whereas 10 were invasive lobular carcinoma (ILC). A total of 448 patients with malignant or borderline disease underwent further surgery after the initial biopsy, among whom 18 patients received unilateral mastectomy and reconstruction; only one paitnet received bilateral mastectomy and reconstruction. The remainders of the patients had neo-adjuvant chemotherapy or salvage first-line chemotherapy depending on the disease.

### Breast measurements, volume and ptosis in Chinese patients

**Fig 1:**
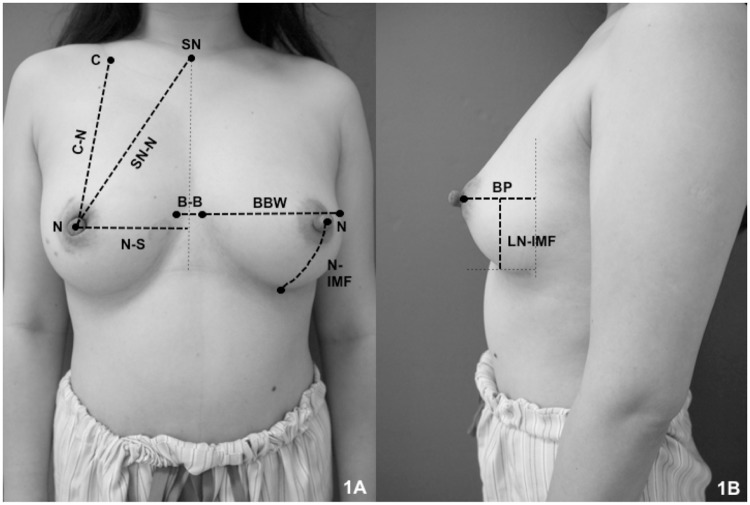
The methodology used to obtain breast measurements (front and lateral views).

The measurement results obtained for 605 patients are listed in Table 2. A description of the methodology used to obtain the breast measurements is illustrated in Table 2 and Fig 1. Notably, the nipple of the Chinese women was situated 10.0±1.5 cm from the mid-sternal line. Thus, the average distance between two nipples was 20.0 cm. The distance from the nipple to the inframammary fold was 7.5±1.6 cm. The width of the breast base was 14.3±1.4 cm (8.5–23.5 cm). The areolar diameter was 3.8±1.0 cm in the horizontal plane and 3.7±1.1 cm in the vertical plane. The nipple diameter was 1.3±0.3 cm with a projection of 0.9±0.3 cm. Among 1210 breasts, 39 (3.2%) had congenital nipple retraction.

**Table 2:** Breast measurement methodology and data for 605 patients.

Parameters	Description	No.	Mean±SD	Range
CCA, cm	Chest circumference under the axilla	605	86.9±6.2	70.0–110.0
CCN, cm	Chest circumference across the nipple	605	89.6±8.4	65.0–122.0
B-B, cm	The distance between the medial rear of both breasts	599	1.75±0.6	0.3–4.8
SN-N, cm	Sternal notch to nipple distance	1196	21.6±2.8	12.5–34.5
C-N, cm	The mid-point of clavicle to nipple distance	1202	22.6±3.1	12.3–35.5
N-IMF, cm	Nipple to the mid-point of infra mammary fold	1182	7.5±1.6	4.0–17.0
SN-IMF, cm	N-IMF when breast skin is stretched	1199	9.1±1.7	4.5–18.5
BBW, cm	The horizontal distance from the lateral to the medial rear of the breast	1207	14.3±1.4	8.5–23.5
N-S, cm	The horizontal distance from the nipple to the midline	1209	10.0±1.5	4.0–14.1
BP, cm	The vertical distance from the chest on the anterior axillary line to the highest point of the breast mound	1205	10.2±2.0	3.0–19.4
ADH, cm	Areolar diameter on the horizontal level	1210	3.8±1.0	1.1–8.6
ADV, cm	Areolar diameter on the vertical level	1210	3.7±1.1	1.1–8.3
NP, cm	Nipple projection	1210	0.9±0.3	0.0–2.1
ND, cm	Nipple maximum diameter	1210	1.3±0.3	0.0–2.9
LN-IMF, cm	The vertical distance from the nipple to the infra mammary fold on the lateral view	1208	2.6±2.9	(-9.1)-8.1

SD, standard deviation; CCA, chest circumference under the axilla; CCN, chest circumference across the nipple; SN-N, sternal notch-nipple distance; C-N, clavicle-nipple distance; N-IMF, nipple-inframammary fold distance; SN-IMF, stretched nipple-inframammary fold distance; BBW, breast base width; N-S, nipple-sternal distance; B-B, breast-breast distance; BP, breast projection; ADH, areolar diameter on the horizontal level; ADV, areolar diameter on the vertical level; NP, nipple projection, ND, nipple diameter; LN-IMF, nipple-inframammary fold distance on the lateral view (LN-IMF)

**Fig 2:**
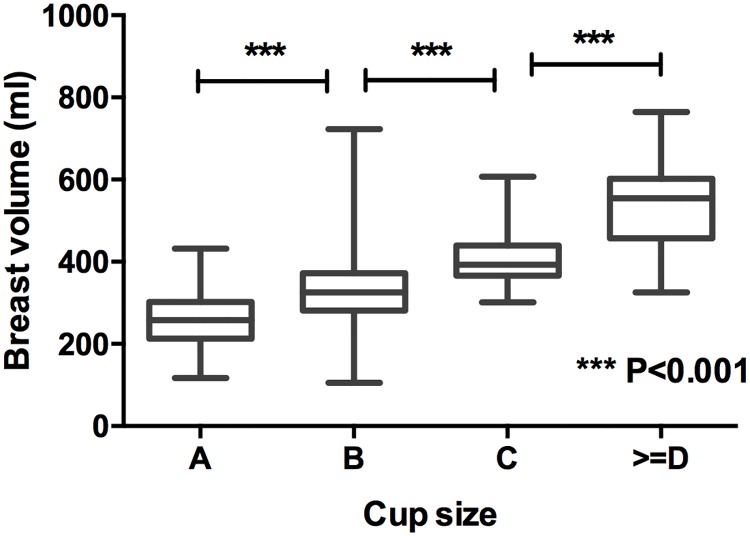
The correlation of breast volume with breast cup size.

Of 605 patients, only 278 women were aware of their bra cup size: 78 (28.1%) reported to be an A cup, 142 (51.1%) were a B cup, 44 (15.8%) were a C cup, nine (3.24%) were a D cup, four were an E (1.4%) cup and one (0.4%) was an F cup. We then calculated the breast volume of the women according to the formula described above (the modified formula of BREAST-V.). The average breast volume was 340.0±109.1 ml (range: 91.8–919.2 ml). The average breast volume for A, B, C and ≥D cups was 260.9±7.9 ml, 328.0±6.9 ml, 408.1±11.3 ml, and 539.0±34.2 ml, respectively (P<0.001, Fig 2).

Based on the RP scale, 274 of 1204 (22.8%) breasts had ptosis, among which 145 (52.9%) were grade I, 92 (33.6%) were grade II and 37 (23.5%) were grade III. According to the LH scale, the nipples of 65 (26.1%) breasts were located within 1 cm below the inframammary fold, 105 (42.2%) were located ≥1 cm but <3 cm below the inframammary fold, and 79 (31.7%) were located ≥3 cm below the inframammary fold. There were 6 and 2 missing data points for two methods.

### Correlation between demographic factors of Chinese patients and breast measurements

Univariate (S1 Table) and multivariate ordinal logistic regression analyses were conducted to explore the correlation between demographic factors and breast volume and ptosis. In a multivariate ordinal logistic regression model (Table 3), every 5 cm of increased height was correlated with an increased breast volume by each quartile (OR[odds ratio] = 1.500, 95% CI 1.259–1.786, *P*<0.001). Post-menopausal status (OR = 1.463, 95% CI 1.062–2.015, *P* = 0.02) and a higher BMI were also positively correlated with an increased breast volume. Breastfeeding for 7–12 months and more than one year were risk factors for an increased breast volume, with a OR of 1.882 (95% CI 1.178–3.007, *P* = 0.008) and 2.367 (95% CI 1.425–3.929, *P* = 0.001), respectively. However, breastfeeding for less than half a year was not associated with an increased breast volume (OR = 1.335, 95% CI 0.767–2.326, *P* = 0.307). Age, literacy, and delivery were not significant in the multivariate analysis.

**Table 3:** Multivariate ordinal logistic regression analysis for breast volume.

Parameters	Breast volume		
	OR	95%CI	*P*
Height (every 5cm)	1.500	1.259–1.786	<0.001
Pre-menopause	1.000		
Post-menopause	1.463	1.062–2.015	0.020
BMI<20.7	1.000		
20.7–22.4	1.752	1.144–2.685	0.010
22.5–24.6	3.406	2.196–5.283	<0.001
≥24.7	8.746	5.427–14.094	<0.001
No breastfeeding	1.000		
1–6 months	1.335	0.767–2.326	0.307
7–12 months	1.882	1.178–3.007	0.008
≥13 months	2.367	1.425–3.929	0.001

OR, odds ratio; CI, confidence interval; BMI, body mass index

For breast ptosis, using either the LH or RP scale revealed similar results that post-menopausal status (for the LH scale, OR = 2.390, 95% CI 1.582–3.613, *P*<0.001; for the RP scale, OR = 2.621, 95% CI 1.765–3.893, *P*<0.001), BMI ≥24.7 kg/m^2^ (for the LH scale, OR = 3.149, 95% CI 1.718–5.774 *P*<0.001; for the RP scale, OR = 2.495, 95% CI 1.420–4.384, *P* = 0.002) were independent risk factors (Table 4). Breastfeeding for more than six months was an independent predictive factor for severer ptosis. Notably, 7–12 months of breastfeeding increased the risk for severer ptosis by 4-fold (for the LH scale, OR = 4.136, 95% CI 1.579–10.838, *P* = 0.004; for the RP scale, OR = 4.010, 95% CI 1.667–9.642, *P* = 0.002), whereas ≥13 months of breastfeeding was associated with a 6-fold increase in the risk of ptosis (for the LH scale, OR = 6.934, 95% CI 2.643–18.194, *P*<0.001; for the RP scale, OR = 6.707, 95% CI 2.775–16.208, *P*<0.001) compared with no breastfeeding. However, breastfeeding for 1–6 months was not a predictive factor for ptosis. Age, height, literacy, and number of births were not significant in the multivariate analysis for breast ptosis.

**Table 4:** Multivariate ordinal logistic regression analysis for breast ptosis using LH and RP score.

Parameters	Breast ptosis (LH)			Breast ptosis (RP)		
	OR	95%CI	*P*	OR	95%CI	*P*
Pre-menopause	1.000			1.000		
Post-menopause	2.390	1.582–3.613	<0.001	2.621	1.765–3.893	<0.001
BMI<20.7	1.000			1.000		
20.7–22.4	1.193	0.619–2.298	0.598	0.952	0.515–1.758	0.875
22.5–24.6	1.614	0.862–3.021	0.135	1.425	0.798–2.542	0.231
≥24.7	3.149	1.718–5.774	<0.001	2.495	1.420–4.384	0.002
No breastfeeding	1.000			1.000		
1–6 months	2.545	0.870–7.441	0.088	2.156	0.797–5.831	0.130
7–12 months	4.136	1.579–10.838	0.004	4.010	1.667–9.642	0.002
≥13 months	6.934	2.643–18.194	<0.001	6.707	2.775–16.208	<0.001

LH, LaTrenta and Hoffman’s scale; RP, Regnault P scale; OR, odds ratio; CI, confidence interval; BMI, body mass index

## Discussion

A wide variation has been observed in the size, shape, and breast morphology projection, depending on age, race, weight and hormonal alterations[10,11]. The current study measured breast features in a large cohort of 605 Chinese patients, of whom 458 had breast cancer. Anthropomorphic data are indispensable for achieving symmetry of both breasts during breast reconstruction.

In a previous study conducted by Longo et al., the breasts of 88 Caucasian women undergoing modified radical mastectomy were measured[2]. They reported SN-N as 24.73±3.48 cm, C-N as 24.96±3.15 cm, N-IMF as 9.26±3.20 cm, BBW as 16.65± 4.80 cm, and areolar diameter as 4.79±1.10 cm, compared with 21.6±2.8 cm, 22.6±3.1 cm, 7.5±1.6 cm, 14.3±1.4 cm and 3.8±1.0 cm (horizontal level), respectively in the current study. However, the mean age of the cohort in the study by Longo et al. was 51.3±9.13 years compared with 48 years in the current study, and the anthropomorphic measurements were generally greater than those in our cohort.

In other studies, the breasts of healthy women have been measured and found to be different from the current study in terms of the patient races, measurement methods and definition of parameters. The mean SN-N value was 18.6 cm in the study reported by Westreich et al.[12] in women with aesthetically perfect breasts, 19.1 cm in the study by Qiao et al.[5] in 18-26-year-old Chinese females, and approximately 22.0 cm in the 40-50-year-old group in the study by Kim SJ[11] of premenopausal Korean females. The mean N-IMF value was 8.3 cm in the study by Avxsar et al. of Turkish women aged between 18 and 26 years, and 6.8 cm and 6.0 cm in the studies by Qiao and Kim, respectively. Because these studies mostly admitted healthy young women, they are only applicable for that specific age group of patients.

Several anthropomorphic models had been proposed to predict breast volume. Qiao et al. first reported a formula based on 250 breasts in young Chinese women[5] and reported an average breast volume of 310–330 ml. Nevertheless, the authors did not validate their model with mastectomy specimens, nor did they compare the results with other models. More recently, Longo et al. developed a formula (the BREAST-V) and validated its reliability with mastectomy specimens[2]. Hence, we used a modified formula of BREAST-V to estimate breast volume in the current study and determined an average breast volume of 340.0±109.1 ml. Other studies have suggested an average breast volume of 407.2–623.5 ml in European women[13,14] and 325.4–386.0 ml in Asian women [5,11].

Although breast volume is particularly important for breast surgery, there is no universally accepted standard measurement method. Kayar et al.[14] compared five different methods with mastectomy specimens (mammography, anthropometric, thermoplastic casting, the Archimedes procedure, and the Grossman-Roudner device) and found that the most accurate method was mammography for all volume ranges, followed by the Archimedes method. Most recent studies have suggested that 3D modelling and MRI are the most reliable tools. However, linear measurements and mathematical modelling remain the most economical and efficient techniques, especially in developing regions with limited relevant resources[15].

In the multivariate ordinal logistic regression analysis, we identified height, post-menopausal status, higher BMI, and breastfeeding for more than six months to be positively correlated with a larger breast volume. These results are consistent with previous studies in which overweight patients demonstrated a 20 ml increase in breast volume for each kilogram above their ideal weight[16]. Kim et al. also found a positive correlation between breast volume and both age and weight[11]. By studying the ideal anthropomorphic values of the Caucasian female breast, Liu et al. found that ideal measurements in women with a higher BMI were universally greater, signifying that a slightly larger breast should be constructed in overweight patients to match the larger frame[17].

The breast ptosis measurements provided consistent results for the RP and LH scales of 22.8% and 26.1%, respectively. Thirty-seven patients were rated as grade III ptosis using the RP score compared with 79 using the LH score. For these patients with grade III ptosis, mastopexy mammoplasty should be considered for those who desire oncoplastic breast-conserving surgery and breast reconstruction.

There are several well-established factors that contribute to breast ptosis. Regnault reported that ptosis was related to glandular hormonal regression in post-menopausal females[8]. Rinker B et al. identified older age, history of significant (50 lbs.) weight loss, higher BMI, larger bra cup size, greater number of pregnancies, and positive smoking history as significant risk factors for ptosis[18]. Kim et al. reported that age and parity were significantly correlated with breast ptosis[11].

However, the most debatable risk factor is breastfeeding. As noted in a 2003 survey of American mothers, the rate of breastfeeding 6 months after delivery was 32.8%[19], partly due to the possible correlation between breastfeeding and ptosis. Although some studies have reported a lack of statistical significance between the history/duration of breastfeeding and breast ptosis[11,18], our findings suggest that breastfeeding for more than six months, together with a post-menopausal status and BMI ≥24.7 kg/m^2^, are independent risk factors for ptosis in this cohort of women with breast diseases. Nevertheless, up to 70.1% of women breastfed for more than half a year in our cohort, and only 22%-26% of all the women had mild to severe ptosis. Therefore, breastfeeding should not be discouraged because breast milk provides indisputable health benefits to the infant[20].

There are several limitations of the current study. Firstly, we did not compare the calculated results of breast volume with other algorithms, nor did we validate the modified BREAST-V with the mastectomy specimens. Moreover, we failed to evaluate the correlation between volume measurement and reconstruction decision since only a few patients received breast reconstruction in our cohort. Future studies are awaited to compare breast measurement data with mastectomy specimens as well as post-reconstruction results.

## Conclusions

The current study provides anthropomorphic measurement data of Asian women, particularly those suffering from breast cancer. The average breast volume was 340.0± 09.1 ml calculated by the modified BREAST-V, and 22%-26% of all the women had mild to severe ptosis. Post-menopausal status, increased BMI, and breastfeeding for more than six months were independent risk factors for both increased breast volume and breast ptosis. Unlike breast augmentation and reduction, symmetry is the core aesthetic concept of post-mastectomy reconstruction, and therefore estimations of breast volume, breast projection and breast base width are critical for both implant-based and autologous reconstructions. Breast ptosis should also be evaluated routinely for patients with breast cancer. Patients with grade II-III ptosis are potential candidates for oncoplastic breast-conserving surgery and contralateral mastopexy.

## Supporting information

S1 Table Univariate ordinal logistic regression analysis for breast volume and ptosis scale. (DOCX)

## References

[1] Tepper OM, Karp NS, Small K, Unger J, Rudolph L, Pritchard A, et al. Three-dimensional imaging provides valuable clinical data to aid in unilateral tissue expander-implant breast reconstruction. Breast J. 2008;14:543–550. 10.1111/j.1524-4741.2008.00645.x 19054001

[2] Longo B, Farcomeni A, Ferri G, Campanale A, Sorotos M, Santanelli F. The BREAST-V: a unifying predictive formula for volume assessment in small, medium, and large breasts. Plast Reconstr Surg. 2013;132:1e–7e. 10.1097/PRS.0b013e318290f6bd 23806950

[3] Jagsi R, Jiang J, Momoh AO, Alderman A, Giordano SH, Buchholz TA, et al. Trends and variation in use of breast reconstruction in patients with breast cancer undergoing mastectomy in the United States. J Clin Oncol. 2014;32:919–926. 10.1200/JCO.2013.52.2284 24550418 PMC4876312

[4] Brown RW, Cheng YC, Kurtay M. A formula for surgical modifications of the breast. Plast Reconstr Surg. 2000;106:1342–1345. 11083567 10.1097/00006534-200011000-00018

[5] Qiao Q, Zhou G, Ling Y. Breast volume measurement in young Chinese women and clinical applications. Aesthet Plast Surg. 1997;21:362–368. 10.1007/s002669900139 9299007

[6] Sigurdson LJ, Kirkland SA. Breast volume determination in breast hypertrophy: an accurate method using two anthropomorphic measurements. Plast Reconstr Surg. 2006;118:313–320. 10.1097/01.prs.0000227627.75771.5c 16874195

[7] Zhang B, Song Q, Zhang B, Tang Z, Xie X, Yang H, et al. A 10-year (1999 ~ 2008) retrospective multi-center study of breast cancer surgical management in various geographic areas of China. Breast. 2013;22:676–681. 10.1016/j.breast.2013.01.004 23391660

[8] Regnault P. Breast ptosis. Definition and treatment. Clin Plast Surg. 1976;3:193–203. 1261176

[9] La Trenta GS, Hoffman LA. Breast Reduction Aesthetic Plastic Surgery. 2nd ed Philadelphia: WB Saunders; 1994:971–975.

[10] Sanuki J, Fukuma E, Uchida Y. Morphologic study of nipple-areola complex in 600 breasts. Aesthet Plast Surg. 2009;33:295–297. 10.1007/s00266-008-9194-y 18626683

[11] Kim SJ, Kim M, Kim MJ. The affecting factors of breast anthropometry in Korean women. Breastfeed Med. 2014;9:73–78. 10.1089/bfm.2013.0068 24180472

[12] Westreich M. Anthropomorphic breast measurement: protocol and results in 50 women with aesthetically perfect breasts and clinical application. Plast Reconstr Surg. 1997;100:468–479. 9252618 10.1097/00006534-199708000-00032

[13] Avşar DK, Aygit AC, Benlier E, Top H, Taşkinalp O. Anthropometric breast measurement: a study of 385 Turkish female students. Aesthet Surg J. 2010;30:44–50. 10.1177/1090820X09358078 20442074

[14] Kayar R, Civelek S, Cobanoglu M, Gungor O, Catal H, Emiroglu M. Five methods of breast volume measurement: a comparative study of measurements of specimen volume in 30 mastectomy cases. Breast Cancer (Auckl). 2011;5:43–52. 21494401 10.4137/BCBCR.S6128 PMC3076010

[15] Xi W, Perdanasari AT, Ong Y, Han S, Min P, Su W, et al. Objective breast volume, shape and surface area assessment: a systematic review of breast measurement methods. Aesthet Plast Surg. 2014;38:1116–1130. 10.1007/s00266-014-0412-5 25338712

[16] Strömbeck JO, Malm M. Priority grouping in a waiting list of patients for reduction mammaplasty. Ann Plast Surg. 1986;17:498–502. 3827119

[17] Liu YJ, Thomson JG. Ideal anthropomorphic values of the female breast: correlation of pluralistic aesthetic evaluations with objective measurements. Ann Plast Surg. 2011;67:7–11. 10.1097/SAP.0b013e3181f77ab5 21301308

[18] Rinker B, Veneracion M, Walsh CP. Breast ptosis: causes and cure. Ann Plast Surg. 2010;64:579–584. 10.1097/SAP.0b013e3181c39377 20354434

[19] Ryan AS, Zhou W, Arensberg MB. The effect of employment status on breastfeeding in the United States. Womens Health Issues Off Publ Jacobs Inst Women's Health. 2006;16:243–251. 10.1016/j.whi.2006.08.001 17055377

[20] Dewey KG, Heinig MJ, Nommsen-Rivers LA. Differences in morbidity between breast-fed and formula-fed infants. J Pediatr. 1995;126:696–702. 7751991 10.1016/s0022-3476(95)70395-0

