# Supplementary material for: A prospective study of breast anthropomorphic measurements, volume and ptosis in 605 Asian patients with breast cancer or benign breast disease
Source: PLoS One. 2017 Feb 13;12(2):e0172122. doi: 10.1371/journal.pone.0172122 (PMC5305059; doi:10.1371/journal.pone.0172122)
Supplement: S1 Table — (DOCX) [file pone.0172122.s001.docx]

Supplementary Table 1 Univariate ordinal logistic regression regression analysis for breast volume and ptosis scale.

| Parameters | Breast volume | | | Ptosis (LH) | | | Ptosis (RP) | | |
| --- | --- | --- | --- | --- | --- | --- | --- | --- | --- |
|  | HR | 95%CI | *P* | HR | 95%CI | *P* | HR | 95%CI | *P* |
| Age (every 5 years) | 1.147 | 1.074-1.225 | <0.001 | 1.296 | 1.187-1.415 | <0.001 | 1.332 | 1.222-1.452 | <0.001 |
| Height (every 5cm) | 1.282 | 1.088-1.509 | 0.003 | 0.768 | 0.624-0.947 | 0.013 | 0.756 | 0.619-0.924 | 0.006 |
| BMI <20.7 | 1.000 |  |  | 1.000 |  |  | 1.000 |  |  |
| BMI 20.7-22.4 | 1.797 | 1.186-2.723 | 0.006 | 1.455 | 0.773-2.741 | 0.245 | 1.099 | 0.612-1.972 | 0.752 |
| BMI 22.5-24.6 | 3.416 | 2.227-5.240 | <0.001 | 2.160 | 1.178-3.961 | 0.013 | 1.840 | 1.061-3.192 | 0.030 |
| BMI ≥24.7 | 10.033 | 6.330-15.900 | <0.001 | 4.904 | 2.753-8.735 | <0.001 | 3.915 | 2.310-6.636 | <0.001 |
| ≤ primary school | 1.000 |  |  | 1.000 |  |  | 1.000 |  |  |
| Middle school | 0.904 | 0.643-1.269 | 0.559 | 0.754 | 0.497-1.144 | 0.184 | 0.630 | 0.422-0.940 | 0.024 |
| ≥ college | 0.628 | 0.422-0.934 | 0.022 | 0.440 | 0.255-0.758 | 0.003 | 0.386 | 0.230-0.650 | <0.001 |
| Pre-menopause | 1.000 |  |  | 1.000 |  |  | 1.000 |  |  |
| Post-menopause | 1.887 | 1.401-2.543 | <0.001 | 3.143 | 2.131-4.636 | <0.001 | 3.459 | 2.377-5.034 | <0.001 |
| No delivery | 1.000 |  |  | 1.000 |  |  | 1.000 |  |  |
| 1 delivery | 3.519 | 1.760-7.034 | <0.001 | 2.270 | 0.699-7.375 | 0.173 | 2.626 | 0.804-8.582 | 0.110 |
| ≥ 2 deliveries | 6.036 | 2.921-12.470 | <0.001 | 4.996 | 1.517-16.449 | 0.008 | 6.383 | 1.928-21.126 | 0.002 |
| No breastfeeding | 1.000 |  |  | 1.000 |  |  | 1.000 |  |  |
| 1-6 months | 1.507 | 0.882-2.574 | 0.134 | 2.797 | 0.979-7.992 | 0.055 | 2.484 | 0.930-6.634 | 0.070 |
| 7-12 months | 1.929 | 1.226-3.035 | 0.005 | 4.225 | 1.646-10.849 | 0.003 | 4.193 | 1.755-10.018 | 0.001 |
| ≥13 months | 3.153 | 1.957-5.078 | <0.001 | 9.657 | 3.772-24.723 | <0.001 | 9.475 | 3.966-22.637 | <0.001 |

LH, LaTrenta and Hoffman’s scale; RP, Regnault P scale; HR, hazard ratio; CI, confidence interval; BMI, body mass index
